# FLDQN: Cooperative Multi-Agent Federated Reinforcement Learning for Solving Travel Time Minimization Problems in Dynamic Environments Using SUMO Simulation

**DOI:** 10.3390/s25030911

**Published:** 2025-02-03

**Authors:** Abdul Wahab Mamond, Majid Kundroo, Seong-eun Yoo, Seonghoon Kim, Taehong Kim

**Affiliations:** 1 School of Information and Communication Engineering, Chungbuk National University, Cheongju 28644, Republic of Korea; wahabmamond@cbnu.ac.kr (A.W.M.); kundroomajid@cbnu.ac.kr (M.K.); 2 School of Artificial Intelligence, Daegu University, Gyeongsan 38453, Republic of Korea; seyoo@daegu.ac.kr; 3 MOTOV Co., Ltd., Seoul 06044, Republic of Korea

**Keywords:** federated learning, deep reinforcement learning, SUMO, agents cooperation, travel time minimization

## Abstract

The increasing volume of traffic has led to severe challenges, including traffic congestion, heightened energy consumption, increased air pollution, and prolonged travel times. Addressing these issues requires innovative approaches for optimizing road network utilization. While Deep Reinforcement Learning (DRL)-based methods have shown remarkable effectiveness in dynamic scenarios like traffic management, their primary focus has been on single-agent setups, limiting their applicability to real-world multi-agent systems. Managing agents and fostering collaboration in a multi-agent reinforcement learning scenario remains a challenging task. This paper introduces a cooperative multi-agent federated reinforcement learning algorithm named FLDQN to address the challenge of agent cooperation by solving travel time minimization challenges in dynamic multi-agent reinforcement learning (MARL) scenarios. FLDQN leverages federated learning to facilitate collaboration and knowledge sharing among intelligent agents, optimizing vehicle routing and reducing congestion in dynamic traffic environments. Using the SUMO simulator, multiple agents equipped with deep Q-learning models interact with their local environments, share model updates via a federated server, and collectively enhance their policies using unique local observations while benefiting from the collective experiences of other agents. Experimental evaluations demonstrate that FLDQN achieves a significant average reduction of over 34.6% in travel time compared to non-cooperative methods while simultaneously lowering the computational overhead through distributed learning. FLDQN underscores the vital impact of agent cooperation and provides an innovative solution for enabling agent cooperation in a multi-agent environment.

## 1. Introduction

The traffic congestion problem has received considerable attention in recent years due to its urgency and the rapid development of urbanization. Traffic congestion is defined as the state of traffic flow in which the road capacity is less than the traffic demand. According to a study from the U.S. Department of Transportation [1], traffic congestion is considered common in most metro areas, so the accurate and effective detection of traffic congestion is in huge demand. When traffic congestion is successfully identified, it cannot only help to reduce congestion and minimize travel time but also lead to preventing tiredness from driving from long trips, reducing economic losses, fuel consumption, pollution, and smartly selecting optimal routes. Traffic congestion detection and the vehicle routing problem (VRP) have been studied for decades; traffic congestion is one of the most important problems that is tackled by Intelligent Transportation Systems (ITSs). In recent decades, numerous approaches and methods have been proposed and implemented to reduce traffic congestion, and more recently, ITSs are playing an essential role in improving traffic performance in smart cities [2]. The focus is on promoting the development of transportation systems. Despite the fact that a lot of maps and navigation systems can provide real-time traffic information, which was an early solution to VRP, they rely more on user feedback than on data directly from the road infrastructure. This reliance on user input introduces a delay in the system’s ability to detect and respond to changes in traffic conditions, which may be problematic for the effectiveness of congestion detection and the precision of estimated travel times. Classical vehicle routing problems prioritize discovering the shortest path while often neglecting other important metrics. Although these studies do consider time costs, they heavily rely on prior knowledge of road networks and operate within static environments [3].

Contrastingly, DRL offers a comprehensive approach, merging Deep Learning (DL) and Reinforcement Learning (RL) [4]. Particularly effective in complex, highly dimensional environments, DRL presents exciting possibilities for discovering new solutions without manual intervention. This adaptive approach enables systems to autonomously learn and adapt to their surroundings. While there has been reasonable progress in RL-based solutions for vehicle routing problems, current methods exhibit limitations. Primarily, these RL-based approaches tend to concentrate on single-agent scenarios, deviating from real-life situations that involve multiple cooperating agents. In these methods, individual learning agents typically handle tasks in a decentralized manner. To address the complexities of vehicle route optimization and travel time minimization, a more robust solution involves employing MARL approaches [5]. Importantly, this necessitates efficient cooperation among the learning agents to achieve optimal outcomes in real-world scenarios.

Although MARL has proven successful in dynamic settings, and multi-agent and distributed AI systems have been studied for decades [6], the realm of effective cooperation and knowledge sharing among agents remains relatively unexplored. There is a lack of comprehensive, in-depth, and insightful studies on how to effectively cooperate agents in the MARL scenarios. Considering this challenge, we propose leveraging federated learning (FL) to enhance efficient and privacy-preserved cooperation and knowledge exchange among agents within a MARL setting. Our work introduces an advanced algorithm, Federated Learning-based Deep Q-Learning Network (FLDQN), designed explicitly to train intelligent agents with the primary goal of minimizing the total travel time, which is the time taken by each agent to complete its journey from the source to destination. This algorithm showcases versatility in addressing various combinatorial optimization problems by integrating RL and FL. Specifically, we leverage FLDQN to tackle vehicle routing problems for travel time minimization and decision-making tasks involving multiple agents. Additionally, we seamlessly simulate real-world traffic conditions by integrating intelligent navigation agents with the microscopic traffic simulator, SUMO, effectively incorporating agent decisions for precise and efficient evaluations.

In this study, we specifically define the “travel time minimization problem” as optimizing the overall time for a vehicle to travel between locations in a specified network or transportation system within the broader context of the vehicle routing problem, with the goal of reducing the overall travel time. In order to analyze the travel time minimization problem, we assume that the road network is represented by a directed graph $G=\left(V,E\right)$ that consists of the set of *m* vertices $V=\left\{v_{1},v_{2},\ldots ,v_{m}\right\}$ representing junctions and the set of *k* edges $E=\left\{e_{1},e_{2},\ldots ,e_{k}\right\}$ representing roads. In our scenario, the multiple agents denoted as $\left\{Agent_{1},Agent_{2},\ldots ,Agent_{n}\right\}$ operate within the environment with the goal of learning and choosing optimal actions. Their primary objective is to minimize the risk of traffic congestion and optimize their routes, ultimately striving to reduce the total required travel time.

**Figure 1:**
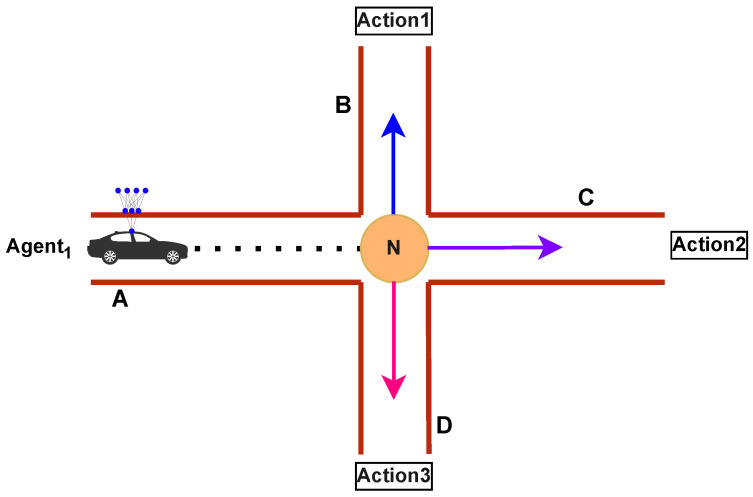
At the intersection N, the agent must choose amongst three options to successfully reach its destination: continuing straight through route C, going left via route B, or going right using route D. The vehicle must determine its route before approaching intersection N.

As an example, let us consider a single agent amongst the agents, denoted as $Agent_{1}$ in Figure 1. As $Agent_{1}$ progresses toward intersection N along the way to its destination, it encounters three potential routes. It must make a critical decision: continue straight through route C, turn left via route B, or head to the right using route D. The challenge arises from the need for the agent to intelligently evaluate traffic conditions along these routes, considering factors such as traffic volume, speed limits, road distance and other important characteristics. The goal is for the agent to make a real-time, informed decision that minimizes traffic congestion and optimizes its route for efficient travel to the destination.

The primary contributions of our research can be summarized as follows:

1. Cooperative Multi-Agent Framework: The cooperative multi-agent framework leverages FL and RL for making a novel approach for solving travel time minimization problems in dynamic environments by enabling multiple agents to work together and share knowledge. The framework can be used in other scenarios where MARL needs efficient and privacy-preserving cooperation mechanisms like ride sharing, fleet management systems, urban tech companies, and autonomous vehicles, where unforeseen conditions require new learned policies.
2. Distributed Training and Centralized Learning: In our approach, agents share their learned policies with a central server, which can lead to improved global model performance, as agents can leverage the strengths of one another. The agents explore the environment independently to hold diverse information or policies and then share them with a federated server, which can improve the global model sequentially.
3. Significant Reduction in Travel Time and in Computational Cost: Our approach is specifically designed to solve the travel time minimization problems in ITSs by leveraging RL and FL, where the travel time is measured for each agent to travel from the starting point to the designated destination, and the computation cost is measured for how fast the agents can learn in the environment. Comprehensive experimental results show that our approach achieves faster and more efficient learning in large scenarios. They also demonstrate that the more agents participate, the better the overall system performs and the shorter the learning duration of the model. Overall, this collaborative process not only enhances the robustness of the model but also accelerates convergence by integrating the varied experiences of different agents.

The subsequent sections of this paper are structured as follows: In Section 2, a concise summary of related studies is described to clarify the motivation of our work. We provide a brief overview of FL, SUMO, RL and DQN in Section 3. In Section 4, we provide the details of proposed framework and introduce the main component of the algorithm. The experimental setup, including the system design, overview, and analysis of the obtained results, along with the architecture of the model are given in Section 5. Finally, we summarize the paper and conclude with future work in Section 6.

## 2. Related Works

A significant amount of research has been conducted for solving VRP. In many classical approaches, the focus is on vehicle path planning and directing vehicles to their destination as soon as possible with the consideration of static conditions, mostly considering distance. Early approaches, like the use of Dijkstra’s algorithm [7], focused on finding the shortest path between nodes in a static environment, lacking consideration for factors such as congestion and speed limits. However, those methods became inadequate as modern transportation networks rapidly developed. The traditional objective of the classical VRP was to minimize a cost function [3], which was considered to be the total distance traveled by all vehicles. Common applications like Google Map and Waze [8] also emerged, aiming to find the shortest path between nodes. Yet, their reliance on human input makes them prone to biases and inaccuracies. In response to these challenges, a Distributed Intelligent Traffic System (DITS) based on ant colony optimization was introduced [9]. Inspired by natural ant behavior in finding food resources, this approach ought to enhance traffic conditions.

The number of VRPs solution methods introduced in the academic literature has grown rapidly over the past few decades. Several notable approaches have been extensively reviewed in the literature as highlighted by [10,11,12,13]; these reviews provide valuable insights into commonly employed methodologies. Kechagiopoulos and Beligiannis [14] introduced an early approach to address the Urban Transit Routing Problem (UTRP), utilizing a particle swarm optimization algorithm to optimize route networks while balancing service quality for passengers and operator costs. Their work demonstrated competitive results using Mandl’s benchmark problem. According to [15], the study of VRPs is growing fast in operations research, increasing by 6% each year. This rapid growth creates a problem for both researchers and practitioners because it is hard to keep up with the latest developments. The large amount of literature makes it difficult to fully understand the newest types of VRPs and how to solve them. Hence, there is a significant demand to systematically organize and summarize information to gain a clearer understanding of the field’s progression. This need arises from the fact that real-world problems involve complex constraints, and advanced algorithms are required to solve VRPs in complicated and constantly changing environments.

The evolution of machine learning (ML) has prompted researchers to investigate Artificial Intelligence-based (AI-based) models for tackling issues related to traffic congestion [16]. To effectively address the challenges posed by VRPs, Bai et al. [17] conducted a thorough review of hybrid methods that combine analytical techniques with ML tools. The use of cutting-edge ML and DL algorithms to solve dynamic VRP is highlighted in [18]. The goal of this research is to continuously improve VRP solving by applying cutting-edge ML and DL algorithms. Conversely, Sabet and Farooq [19] provide a comprehensive review of the Green Vehicle Routing Problem (GVRP). This variant of VRP aims to minimize greenhouse gas emissions in vehicle routing by considering Alternative Fuel Vehicles (AFVs) alongside conventional fossil fuel vehicles in fleets. The overarching goal is to achieve environmental sustainability in transportation and logistics. Furthermore, Li et al. [20] explore the vehicle routing problem as a discrete combinatorial optimization challenge. This study highlights the existing models and algorithms designed for resolving VRPs. Notably, the exploration extends to Learning-Based Optimization (LBO) algorithms as potential solutions for addressing the complexities of VRPs.

The increasing popularity of DL and RL techniques marks a departure from traditional navigation methods. These traditional approaches heavily depend on human input and tend to overlook contemporary infrastructure limitations. In contrast, DL and RL techniques have garnered attention for their ability to offer more advanced and automated solutions in the realm of navigation. As covered in [21], DRL techniques have been proposed to address the problem of traffic congestion and enable intelligent vehicle routing and navigation in urban contexts. Specifically, this approach addresses Stochastic Dynamic Vehicle Routing Problems (SDVRPs), where proactive real-time routing is necessary. The paper explores the application of RL to evaluate these actions, recognizing the challenges posed by the complex and combinatorial action space inherent in SDVRPs. This is a new attempt to apply DRL methods to route planning. The goal of the article, as shown by [22], focuses on developing a route-guiding method capable of handling dynamic traffic situations. The proposed approach leverages RL to solve dynamic route planning problems. Notably, the waiting time before each traffic light is considered a reward factor in the algorithm. Another aspect of route planning is explored in [23], where the emphasis shifts to pedestrians. The study introduces a route planning algorithm based on DRL, predicting pedestrian flow with travel time consumption as the metric. A more comprehensive approach to addressing VRP using RL is presented in [24]. This work introduces an end-to-end framework, primarily concentrating on training a singular model capable of generating near-optimal solutions for problem instances sampled from a specified distribution. Additionally, Wei et al. [25] analyze the spatiotemporal patterns of traffic congestion in 77 large Chinese cities using real-time big data; the study identifies distinct congestion patterns and variations in performance on different days of the week. Integrating RL into ITS is a highly advantageous choice, especially considering the dynamic nature of most environments. The agent’s ability to learn and adapt over time aligns seamlessly with the evolving conditions in such dynamic settings.

Despite the rich history of RL, only a few studies have addressed MARL challenges in ITSs [26,27], and they are facing efficient agent cooperation problems. According to a thorough analysis by DeepMind [28], collaboration problems are important in many different sectors and are ubiquitous in both daily tasks and global challenges. Chu et al. [29] examine the use of RL in complex urban traffic networks to provide Adaptive Traffic Signal Control (ATSC). Akopov et al. [30] address simulation-based optimization by proposing a parallel real-coded genetic algorithm to minimize potential traffic accidents in an Artificial Multi-Connected Road Network (AMCRN).

Recently, several research efforts have explored combining RL and FL. One of the earliest works [31] introduced FedRL, which considers two agents sharing parameters. However, it faces scalability issues and lacked a proper coordinator. In [32], the authors propose FAVOR, focusing on the non-IID nature of client-side data and selecting clients that contribute to global model improvement. The goal of FAVOR is to train the DRL agent to converge to the target accuracy in federated learning as quickly as possible. Soltoggio et al. [6] introduce lifelong learning (LL), an approach that integrates multiple research areas into a collective AI framework capable of learning continuously. While LL can be applied on a large scale (e.g., cross-silo settings), it is not suitable for commercial applications with limited computational power. Li et al. [33] provide a comprehensive overview of the applications of FL in industry, highlighting real-world use cases and suggesting promising directions for future research. It underscores the importance of addressing challenges in FL to improve its practical implementation. Chen et al. [34] address the challenge of the on-ramp merging problem for autonomous vehicles (AVs) in mixed traffic with Human-Driven Vehicles (HDVs). This research formulates the issue as a multi-agent RL problem; they highlight the collaboration and learning policies in a multi-agent setting, but it does not provide specific details on the mechanisms or techniques employed for agents’ cooperation. There are other recently developed approaches that deal with application-based multi-agent systems; Li et al. [35] focus on achieving robust bipartite tracking consensus in second-order multi-agent systems. The proposed approach aims to enhance robustness, reduce computational complexity, and simplify the control system’s structure while managing uncertainties and disturbances in multi-agent systems. Furthermore, Li et al. [36] address a critical problem in the field of control systems, particularly in networked multi-agent systems. The primary goal of the proposed work is to analyze and improve the performance of containment tracking in networked agent systems that are affected by nonuniform communication delays.

To summarize, RL has been widely applied in ITSs and in other application-based scenarios; certain research has shown significant advancements and realistic state assessments. However, benchmark traffic environments are still missing for fair comparisons among different RL algorithms. Additionally, there are very limited previous research findings related to agents’ efficient cooperation in MARL, and the field lacks comprehensive studies focusing on MARL and efficient cooperation among agents. Despite the successful application of DRL for solving traffic and transportation issues, there is a current need for a well-structured network environment to enhance agents’ learning capabilities. Our study, drawing inspiration from comparable studies [37], distinguishes itself by delving into the previously unexplored realm of multi-agent collaboration in-vehicle-navigation. The existing research has overlooked the need for holistic approaches to agents’ cooperation, revealing a substantial gap in the existing literature. Therefore, our study represents the first attempt, to the best of our knowledge, to fill the void in the literature. Previous works have not presented efficient solutions for agents’ cooperation, leaving the challenge unaddressed in the context of vehicle navigation. Note that our proposed objective is more flexible, as it can be used for any scenarios where multiple agents need efficient and privacy-preserved cooperation.

## 3. Preliminaries

This section covers the fundamental concepts that are essential for understanding our scenario. It lays the foundation for a deeper exploration of the specific details we will delve into, starting with the FL, followed by the SUMO simulation and the DQN method we employ.

### 3.1. Federated Learning

Federated learning (FL) [38] is a distinctive form of distributed learning that excels in scenarios where training on the device provides a distinct advantage over training on proxy data at a central data center. This is particularly applicable when dealing with large datasets compared to the model size or sensitive data that are better kept private, leading to the decision not to transmit such data to the central data center. The goal is to avoid storing massive volumes of data on a centralized server or cloud by having machines cooperatively learn a shared prediction model while retaining all the training data on the machines themselves [39]. In the FL paradigm, a server collects the learned parameters periodically to update the global model, which is then distributed back to the clients (agents) for local training and inference. In the server, the local models are aggregated by weighting each local model by the number of available training samples, ensures a collective improvement in the global model while preserving the decentralized nature of the learning process as depicted in Equation (1), where $W_{t+1}$ are the central model parameters, *K* is the sample of all participants, $n_{k}$ is the sample of participants *k*, and $w_{t+1}^{k}$ is the local model parameters of all *k* participants:

(1) $$\;W_{t+1}\leftarrow \sum_{k=1}^{K}\frac{n_{k}}{n}w_{t+1}^{k}$$

### 3.2. SUMO

Simulation of Urban Mobility (SUMO) [40] is an open-source, highly portable, microscopic, and continuous multi-modal traffic simulation system designed to handle large traffic networks. SUMO is a comprehensive and open-source traffic simulation suite that has been freely available since 2001 [41]. SUMO comes with an outstanding ability to simulate a very large and complex transportation network of up to 1000 edges (roads). SUMO is pre-configured with a number of auxiliary tools that are intended to make creating, running, and assessing traffic simulations easier. Multiple APIs for remote simulation control are provided by SUMO, along with the flexibility to integrate custom models. One of these APIs is the Traffic Control Interface (TraCI), which we utilize in our study. TraCI is integrated with SUMO, which allows obtaining information about the current state of the object and changing its state. TraCI enables real-time simulation interaction by giving users the ability to control and monitor how cars, traffic lights, and other simulation elements behave while the simulation is running. TraCI uses a TCP-based client/server architecture, where SUMO acts as a server and the agents as clients. The TraCI client can transmit commands to the SUMO environment, such as starting the simulation or controlling the vehicle movement. SUMO executes the command and returns the result.

SUMO also offers another crucial feature known as the LaneAreaDetector, which is used to monitor and analyze the flow of vehicles within a designated area along one or more lanes. It is similar to a camera used for tracking vehicles but with specific attributes and functionalities tailored for simulation purposes. They are particularly useful for measuring queues of stationary or congested vehicles and keeping track of all vehicles within their designated area. In our scenario, a LaneAreaDetector is deployed on all edges to monitor the vehicle count within the lane. SUMO also provides another useful feature called randomTrip that is designed to generate a series of random trips within a specified network. This feature proves to be instrumental in our work, allowing us to leverage the capability of generating random trips for comprehensive testing and analysis within the SUMO simulation environment.

### 3.3. DQN

**Figure 2:**
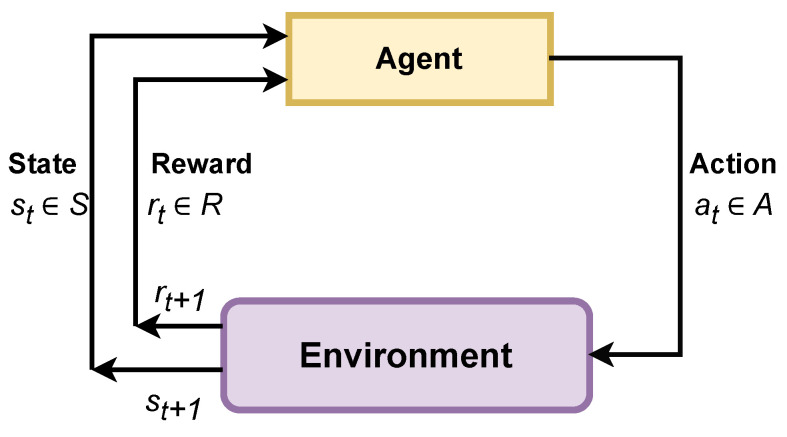
An agent takes action $a_{t}\in A$ in the environment, receiving reward $r_{t}\in R$ and transitioning to the next state $s_{t+1}$ based on the actions taken.

RL [42] is a branch of machine learning that aims to instruct agents to make optimal decisions by interacting with their environment. Rather than being given clear instructions, in RL, an agent learns to make decisions by interacting with an environment. The agent receives feedback in the form of rewards based on the actions it takes in different states of the environment as depicted in Figure 2.

The core components of every RL-based system include the following:

**Agent:** The decision-maker or learner who interacts with the environment and makes decisions based on its current state. The ultimate goal of the agent is to optimize its actions to maximize cumulative rewards within the given environment.

**Environment:** The system outside of the agent with which it communicates, the environment, gives feedback to the agent based on the actions taken; this feedback typically comes in the form of rewards or penalties.

**State:** This is a representation of the current condition in the environment at a specific time. The agent relies on the state to inform its decision-making process and select appropriate actions.

**Action:** This is a decision or choice made by the agent in the environment. The choices of actions are guided by the agent’s policy, which is a set of guidelines and rules. The agent continuously improves its policy through learning to make better decisions over time.

**Reward:** In the realm of RL, the concept of reward holds the utmost significance. It serves as a pivotal motivator for agents, compelling them to converge towards an optimal policy π by reinforcing favorable actions, which are approximated through a specific function.

The primary objective of an agent within this framework is to discern an optimal behavioral policy. This optimal policy aims to maximize the expected long-term discounted reward, denoted as $R_{t}=\sum_{i=t}^{T}\gamma^{i-t}r_{i}$, where *T* signifies the termination step, $R_{t}$ represents the total discounted reward from time-step *t* until the termination step *T*, $r_{i}$ represents the immediate reward at time-step *i*, and γ∈[0,1] represents the discount factor. This discount factor strategically balances the importance of immediate rewards against those in the future.

One of the prominent algorithms in RL is the Deep Q Network (DQN) [43]. It combines deep neural networks with Q-learning to learn the optimal policy for a given task. Q-learning is a method that uses a Q-table of state–action values, also called Q-values [44]. DQN replaces the Q-table with a deep neural network to represent the Q-function, which allows it to learn complex relationships between states and actions. DQN typically uses two neural networks: a main network and a target network. The main network is used to estimate the Q-values for the current state and action. It is a standard neural network that takes the current state as input and outputs the Q-values for all possible actions. The Q-values are then used to select the action with the highest expected reward, as denoted in Equation (2), where $Q_{main}$ denotes the weight matrix of the main network, and $\phi \left(s\right)$ is the feature vector representation of state *s*. While the target network is used to estimate the Q-values for the next state, it is a copy of the main network, and its weights are updated less frequently as denoted in Equation (3), where $Q_{target}$ signifies the weight matrix of the target network and $s^{\prime }$ represents the feature vector representation of state $s^{\prime }$:

(2) $$Q_{main}\left(s,a\right)=W_{main}.\phi \left(s\right)$$

(3) $$Q_{target}\left(s^{\prime },a\right)=W_{target}.\phi \left(s^{\prime }\right)$$

The weight parameters of the main network are duplicated in the target network. This transfer of knowledge from one network to another contributes to more accurate estimations produced by the target network. This helps to stabilize the learning process and prevent the DQN from learning unstable or overestimated Q-values. During the learning process, DL minimizes the error estimated by the loss function by optimizing the weights θ. To compute the loss, Mean Squared Error (MSE) is used to find the difference between the target Q-value and the predicted Q-value as denoted in Equation (4):

(4) $$\mathrm{Loss}=\mathrm{MSE}\left(\mathrm{Predicted}\;Q\text{-}\;\mathrm{value},\;\mathrm{Target}\;Q\text{-}\;\mathrm{value}\right)$$

(5) $$L\left(\theta \right)=E[{\left(r+\gamma max_{a^{\prime }}Q_{target}\left(s^{\prime },a^{\prime };\theta^{\prime }\right)-Q_{main}\left(s,a;\theta \right)\right)}^{2}]$$

In Equation (5), θ is the parameter of the main network, $\theta^{\prime }$ is the parameter of the target network, $Q_{main}\left(s,a;\theta \right)$ is the Q-values predicted by the main network, $Q_{target}\left(s^{\prime },a^{\prime };\theta^{\prime }\right)$ is the target Q-value predicted by the target network for the next state $s^{\prime }$ and the action $a^{\prime }$ that maximizes the Q-value, and (0<γ≤1) is a discount factor that tells us how important future rewards are to the current state. Another crucial component and technique of DQN is known as replay memory, also referred to as experience replay. This technique enhances the learning process by storing the agent’s experiences at each time-step. The agent’s experience at each time-step is represented as a tuple as depicted in Equation (6):

(6) $$e_{t}=\left(s_{t},a_{t},r_{t}\left(s_{t},a_{t}\right),s_{t+1}\right)$$

(7) $$D\left.=e_{1},\ldots ,e_{N}\right.$$

Here, $s_{t}$ represents the state at time *t*, $a_{t}$ is the action taken, $r_{t}\left(s_{t},a_{t}\right)$ is the corresponding reward given to the agent at time *t* as a result of the previous state–action pair $\left(s_{t},a_{t}\right)$, and $s_{t+1}$ is the next state. In Equation (7), D is the dataset where we store the agent’s experiences at each time-step, pooled over many episodes into replay memory. This tuple indeed gives us a summary of the agent’s experience at time *t*. A key reason for using replay memory is to break the correlation between consecutive samples. If the network is learned only from consecutive samples of experience as they occurred sequentially in the environment, the samples would be highly correlated and would therefore lead to inefficient learning; randomizing the samples breaks these correlations and therefore reduces the variance of the updates [43].

## 4. FLDQN

**Figure 3:**
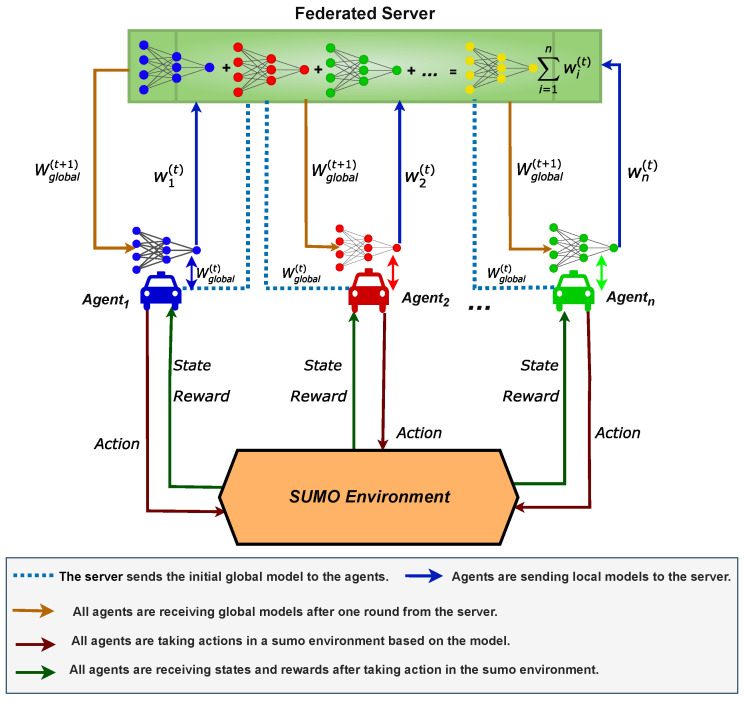
The proposed architecture of FLDQN involves a federated server that coordinates the actions of multiple agents in an SUMO simulation environment. The agents operate within the SUMO environment. After individually training models in the SUMO environment, each agent transmits its trained model back to the server for subsequent processing.

In this section, we delve into the FLDQN algorithm, a powerful approach that fosters collaboration among agents through FL, addressing the challenge of minimizing travel time. A comprehensive overview of FLDQN is illustrated in Figure 3, featuring a federated server, SUMO environment, and agents. The federated server acts as the orchestrator for the FL process [38], overseeing the aggregation of model weights from various agents to facilitate collaboration. Currently, it operates as a central server in the cloud, focusing on controlling the weights exchange for connected intelligent vehicles, but it can also be deployed as a Roadside Unit (RSU) or base station depending on the scenario requirements. SUMO serves as a tool for generating realistic traffic during training.

The agents navigate towards their respective destinations while minimizing the total travel time. Throughout their journeys, each agent refines its DQN model to take optimal actions, leading to higher rewards, which is lower travel time in our scenario. Additionally, in the FLDQN algorithm, agents do not consider distances from other agents but only explore the environment to make actions optimal for maximizing individual rewards, which ultimately contributes to the cumulative reward.

### 4.1. Architecture

In this subsection, we delve into a detailed explanation of the FLDQN algorithm, explaining each key aspect and component. Figure 3 illustrates the architectural diagram for a clearer understanding.

1. **Federated Server:** The federated server initiates the process by distributing an initial global model $W_{global}^{\left(t\right)}$ to all connected agents. Each agent will receive $W_{global}^{\left(t\right)}$ and start training the model on local data, after local training, each agent transmits their updated weights $w_{i}^{\left(t\right)}$ back to the federated server. After receiving the local updated weights from all the connected agents, the server then averages these local updated weights to construct an updated global model $W_{global}^{\left(t+1\right)}$. This iterative process continues until predefined criteria are met. This weight-sharing approach allows agents to undergo simultaneous training by leveraging the collective experience of all agents, even though each may have distinct observations. Throughout training, each agent utilizes its own DQN model, evolving its own hidden states $S_{t}$, making independent action selections, and communicating solely through the federated server. This distributed approach ensures autonomy for each agent while contributing to the cooperative behavior of the entire system. This promotes knowledge sharing and cooperation, aiming for higher rewards $R_{t}$ compared to non-cooperative approaches.
2. **Agents:** Agents are SUMO-based intelligent vehicles equipped with a DQN model. They autonomously take actions based on dynamic road and traffic conditions (states). The role of agents is to train their DQN models and frequently send these model updates to the federated server.
3. **SUMO:** The SUMO simulator serves as the environment for generating realistic traffic scenarios. In our scenario, SUMO facilitates the creation of the road network traffic and provides an interface for interaction with the environment using Python APIs.
4. **States:** States are crucial in any RL-based approach, serving as input to the algorithm during the training phase. In our case, the state space at any time-step *t* is represented as
  $$S_{t}=\left\{N_{veh},\;A_{speed},\;L_{road},\;A_{loc},\;A_{des}\right\}$$
  This representation consists of five elements, each serving a specific purpose:
  - $N_{veh}$: Number of vehicles on the current road.
  - $A_{speed}$: Maximum allowed speed.
  - $L_{road}$: Length of the current road.
  - $A_{loc}$: Current position of the agent.
  - $A_{des}$: Final destination of the agent.
5. **Reward:** FLDQN employs a unique reward mechanism, which is denoted in Equation (8):
  (8) $$R_{s_{t}}=-\left(T_{s_{t+1}}-T_{s_{t}}\right)$$
  Here, an agent receives a total reward, $R_{s_{t}}$, for executing a specific action or reaching a particular state at a given moment. Additionally, $T_{s_{t+1}}$ denotes the total travel time to state $s_{t+1}$, while $T_{s_{t}}$ indicates the total travel time to state $s_{t}$. This approach differs from traditional methods of setting rewards in sequential decision-making processes, which can suffer from long delays. This reward mechanism aims to address this issue, and as a result, it significantly improves the convergence performance of the FLDQN as compared with other discounted reward schemes.

### 4.2. Algorithm Description

This subsection introduces and explains the proposed algorithm, FLDQN (Algorithm 1). The FLDQN is a distributed RL approach designed to foster collaboration among multiple agents, enabling them to learn from shared experiences and enhance decision-making. The algorithm unfolds in two main phases: server- and agent-side execution. In the foregoing discussion, we delve into the specifics of each phase:

**Server side:** In FLDQN, the server plays a crucial role in coordinating the training process and facilitating communication between agents. On the server side, denoted in lines 1 to 8 of Algorithm 1, the global model $W_{global}^{\left(t\right)}$ is shared with all agents through the UpdateAgent() function. This initializes a baseline for all connected agents, ensuring consistent starting points. The server orchestrates the synchronization of local model updates by waiting for all agents to submit their changes. Upon receiving these updates, the server aggregates and averages them, creating the new global model $W_{global}^{\left(t+1\right)}$. This process, known as aggregation, fosters collaboration and knowledge sharing among agents. Subsequently, the updated global model $W_{global}^{\left(t+1\right)}$ is broadcast back to all agents, maintaining consistency for future learning rounds.    
**Algorithm 1:** FLDQN algorithm
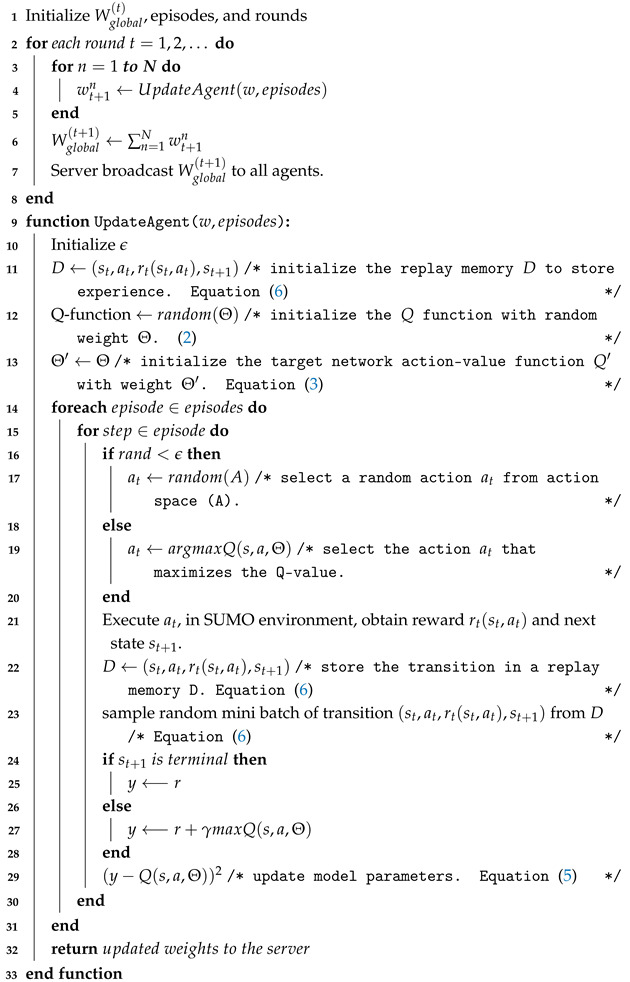


**Agent side:** The UpdateAgent() function (lines 9–33) is executed independently by each agent and takes two parameters, “*w*” and “*episodes*” as input. Here, “*w*” represents the global model parameters received from the server, and “*episodes*” indicates the number of iterations that an agent will undergo in each round.

During the initialization phase (lines 10–13), the agent first initializes the ϵ, which is used later to determine the actions taken by the agent. Then, the replay memory to store experiences is initialized (line 11). The Q-function is initialized to set action values with random weights using Equation (2) (line 12). Next, the agent initializes the target network to provide stable target values during training; $\Theta^{\prime }$ is the weight of the target network and Θ is the weight of the main network, and it indicates to set the weight of the main network to the target network using Equation (3) (line 13). The actual training starts at line 14, where an agent iterates over the episodes and each episode consists of multiple steps. During each step a random value is generated and compared with ϵ. If the generated random value is smaller than the epsilon ϵ, the agent will perform a random action to explore the environment (line 17); otherwise, the agent will perform an action that is suggested by the Q-network (line 19). The selected action is executed in the SUMO environment (line 21), where the agent obtains the reward $r_{t}$ and the next state $s_{t+1}$ based on the action taken. The agent then stores the transition $\left(s_{t},a_{t},r_{t},s_{t+1}\right)$ in the replay memory *D* (line 22). Subsequently, the agent randomly samples a mini-batch of transitions from the replay memory *D*. The replay memory consists of the agent’s experiences: the agent selects a sample mini-batch of transition from *D* to optimize the neural network, and a sample mini-match is randomly selected to remove the correlation between recently stored records and old records (6) (line 23).

The target calculation is performed next. If the next state $s_{t+1}$ is terminal, also known as the final state, meaning the episode ends after this state, then target *y* is set to the observed reward *r*. In other words, reaching the terminal state indicates that there is no future reward for the agent to look forward to, so the target value *y* for updating the Q-function is simply the reward *r* received at this step (line 25). If $s_{t+1}$ is not terminal, then the target *y* is calculated as the sum of the immediate reward *r* and the discounted maximum Q-value of the next state (line 27). This approach incorporates the expected future rewards into the current update. Finally, the Q-function updates the parameter Θ to minimize the loss between the predicted Q-values and the target values *y* using Equation (5) (line 29). After completing the episodes, the updated local model weights are returned to the server for aggregation (line 32).

In FLDQN, each agent refines its policy based on its experiences, thereby improving its decision-making capabilities. The server aggregates the local updates from all agents, ensuring that the global model benefits from diverse experiences. FLDQN allows agents to collectively learn and improve their performance over time, leveraging shared knowledge without sharing raw data. This innovative approach enhances the robustness and generalization of the learned policies, enabling agents to make better decisions in dynamic and complex environments.

## 5. Performance Evaluation

This section evaluates the comparative performance between DQN and FLDQN within the SUMO simulation environment. The assessment will focus on the key performance metrics, including travel time, running time, and the effect of the number of agents in FLDQN, where the running time is defined as the duration required to execute experiments and specific episodes. By analyzing these aspects, we aim to provide a detailed understanding of how FLDQN performs in optimizing travel time and running time in the SUMO environment.

**Evaluation Setup:** FLDQN is implemented using Python v3.8, Flower v1.4.0 [45] is used for FL, and TensorFlow v2.12.0 [46] serves as the underlying Deep Learning library. For simulation purposes, Eclipse SUMO Version v1.14.1 [40] was utilized. In our experiments, five agents were deployed, with a federated server, serving as the central point for training and coordination. All experiments were conducted on a machine equipped with an Intel i7 processor and 64 GB of RAM. To ensure consistent and reliable results, each experiment was run for three trials with different random seeds while keeping other hyperparameters the same across all the trials for both DQN and FLDQN. The final values were taken as the average of the values of the three trials. Due to the large number of episodes (5000), significant fluctuations in performance were observed. To address this, a moving average with a window size of 100 was applied to smooth the results. The detailed hyperparameters used can be found in Table 1.

**Model Architecture:** We employ a five-layer feed-forward neural network with three hidden layers, one input layer, and one output layer. The activation function used throughout the hidden layers is the Rectified Linear Unit (ReLU) [47]. The first dense layer has 150 units, the second dense layer has 100 units, and the third dense layer, using linear activation, has the same number of units as there are actions in the environment. The architecture employs a dueling network structure, characterized by two streams of fully connected layers: the advantage stream and the value stream. The advantage stream, which represents all of the connected roads in the urban network, generates an equal number of outputs as actions. The value stream, in contrast, has a single output. In terms of hyperparameters, for DQN, we set the number of episodes to 5000.

For FLDQN, configured for FL setting with a round concept [48], the number of episodes is set to 100 per round with a total of 50 rounds, which is equivalent to a total of 5000 episodes. These hyperparameters provide the framework for training and evaluating the model’s performance. All the hyperparameters that we used in our experiments are shown in Table 1. Here, “Fraction-fit” determines the fraction of available agents used for model updates, set to 1.0 for full participation. “Min-fit-clients” specifies the minimum number of agents required for model fitting or training, set to 5. “Min-available-clients” sets the minimum number of available clients needed for FL rounds, set to 5, “Episodes” defines the number of training episodes per learning iteration, set to 100. “Rounds” specifies the number of FL rounds, set to 50. “Discount-factor” is set to 0.99. “Learning-rate” defines the step size for updating the model parameters during training, set to 0.001. “Epsilon” controls the exploration-exploitation trade-off in the learning process, set to 1.0. “Epsilon-Decay” determines the rate at which epsilon (exploration factor) decreases over time, set to 0.95. “Epsilon-min” specifies the minimum value for epsilon during exploration, set to 0.05. “Batch-size” sets the number of samples used in each iteration of model training, set to 32. “Train-starter” defines the episode at which training begins, set to 200, and “Reply-Memory” used to store the agent experiences, is set to 100,000.

**SUMO Simulation Environment:** As highlighted in our preliminary section, our study is evaluated through SUMO with smart navigation agents to create a unified experimental environment. SUMO supports the seamless simulation of real-world traffic conditions while concurrently incorporating the decision-making processes of the agents. It also provides two primary methods for network creation: using OpenStreetMap or utilizing the graphical network editor NetEdit. Our network is constructed using the NetEdit method, where we import network and demand traffic XML files, defining the network’s topology and geometry.

**Figure 4:**
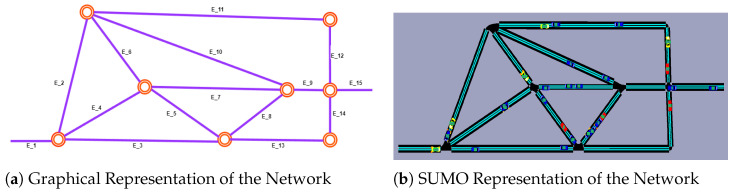
Figure (**a**) is graphical representation of our scenario. We see the actual network operating in the SUMO simulation in Figure (**b**). Two types of vehicles are populated in the simulation: yellow ones represent our agent vehicles; red and blue ones are the randomly generated buses and normal cars.

In Figure 4, we illustrate the graphical scenario (Figure 4a) alongside the corresponding network visualized within the SUMO simulator (Figure 4b). Our network consist of 15 edges, each with distinct properties such as speed limit and length. It is noteworthy that SUMO categorizes vehicles into two types. The first consists of randomly generated vehicles, following a specific probabilistic approach based on Origin–Destination (OD) points to simulate dynamic traffic congestion. These vehicles are marked as red and blue in Figure 4b, representing trucks and regular cars, respectively. The second group comprises agent vehicles operating within the environment with the purpose of learning and selecting optimal actions. These agent vehicles are represented as yellow in Figure 4b. To simulate real-time traffic conditions, agents start from various locations but share a common destination, denoted as $E_{15}$ in Figure 4a. Additionally, we utilize SUMO’s randomTrip.py feature to generate random traffic within the network, specified as [b−0−e−3600−period−3], which indicates that the simulation will run for 3600 s, generating a random vehicle every 3 s until the simulation ends. The simulation involves a total of 1200 non-intelligent vehicles and 5 intelligent agents. This setup ensures realistic traffic conditions while providing a controlled environment for evaluating the performance of intelligent agents.

To validate the practicality of the FLDQN algorithm, we conduct several experiments. In each training step, the framework acquires environmental observations from SUMO and forwards them to the DRL agent. The DRL agent, based on these observations, evaluates traffic conditions and uses its policy neural network to make decisions. The algorithm then executes these actions, updating the state and progressing in SUMO until the simulation concludes. At the end of each simulation run, the reward is calculated and sent to the DRL agent for optimization.

### 5.1. Travel Time Comparison

**Figure 5:**
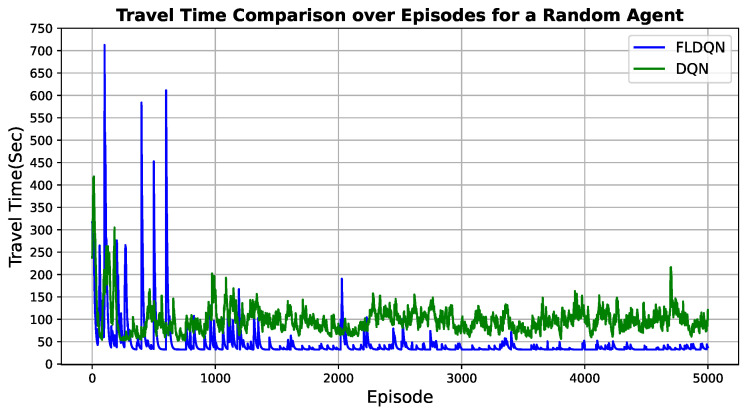
Comparison of travel time for a randomly selected agent: FLDQN achieves a significant reduction in travel time to 46.47 s, surpassing the DQN (98.52 s) under identical map and random traffic conditions.

This subsection evaluates the travel time for both DQN and FLDQN. We utilize the same map and random traffic conditions discussed in the previous section. The evaluation involves 5 agents and 1200 randomly generated vehicles. The travel time for each agent is measured from their starting point to their designated destination. As previously mentioned, we set 5000 episodes for DQN and 100 episodes for FLDQN. In FLDQN, we introduce rounds, with 50 rounds specified, resulting in a total of 100 (episodes) × 50 (rounds) = 5000 episodes for FLDQN. To observe the changes in travel time across 5000 episodes, we compare the travel time of a randomly selected agent as illustrated in Figure 5.

The evaluation results presented in Figure 5 indicate distinct behaviors between DQN and FLDQN. DQN exhibits continuous fluctuations, with some episodes taking over 200 s between 4000 to 5000. In contrast, FLDQN experiences more pronounced fluctuations during its initial 1000 episodes, followed by stabilization efforts from episodes 1000 to 2000. Subsequently, the agent learns and converges to a travel time of less than 100 s from episode 2000 up to 5000.

This initial inconsistency and variability can be attributed to the “Train-starter” hyperparameter (as specified in Table 1), which dictates that agents should explore the environment before initiating their model training. This exploration phase is designed to enhance the cumulative reward in the long term. Since the agents are taking random actions, they are not receiving the expected rewards due to the lack of optimal actions during the initial episodes. Additional communication rounds are imperative for the models to converge through shared updates. However, after sufficient global aggregation steps, beyond 2000 episodes, FLDQN accrues the benefits of model cooperation with other agents, evidenced by the drastic performance improvement. The stabilization and rapid descent to low travel times demonstrate the ability of FLDQN to effectively learn collaborative behaviors. In contrast to the performance improvements seen in FLDQN, the continued instability of the standard DQN algorithm over episodes underscores the advantages of using FL approaches compared to standalone RL agents.

In summary, FLDQN enables agents to exchange their learned experiences by aggregating updates across decentralized models, while DQN utilizes individual RL agents. This results in FLDQN achieving quicker progress towards optimal travel time after an initial divergence, facilitating accelerated learning compared to DQN.

### 5.2. Effects of the Number of Agents

**Figure 6:**
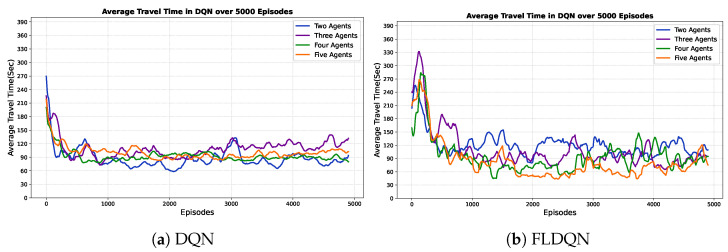
Figure (**a**) illustrates individual agent learning in DQN, where agents independently shape their performance over episodes. On the other hand, Figure (**b**) shows how the number of agents in FLDQN affects how cooperatively they work together and how this correlates with system performance as a whole.

**Figure 7:**
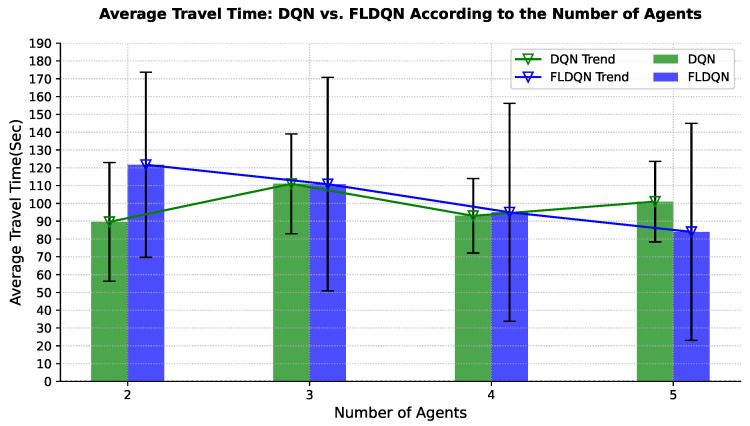
Comparison of average travel time between DQN and FLDQN by varying numbers of agents. Performance in the system is dependent on the number of agents.

This subsection explores how the number of agents influences the performance and dynamics of both DQN and FLDQN. For effective route navigation and optimal travel time, a comprehensive understanding of traffic dynamics is essential. To assess the impact of the number of agents on performance, we conduct experiments with varying agent counts. Our experiments assess the impact on travel time, crucial for effective route navigation. The results in Figure 6 and Figure 7 depict the average travel time over the episodes and number of agents, respectively. DQN agents over episodes, shown in Figure 6a, operate independently without influencing each other throughout the episodes. Clear fluctuations and independence are observed, with each agent running and learning autonomously. For instance, when the number of agents is 2, the average travel time is approximately 89 s, increasing to 111 s for 3 agents, decreasing to 93 s for 4 agents, and again increasing to 101 s for 5 agents over 5000 episodes, showcasing the fluctuation and independence of agents in DQN. Figure 6b emphasizes the results of our proposed FLDQN approach, highlighting agent cooperation and the significant impact of the number of agents on overall system performance. In FLDQN, the number of agents affects the overall system performance as depicted in Figure 6b; for instance, when the number of agents is set to 2, it takes 121 s for 5000 episodes, decreasing to an average of 110 s for 3 agents, 95 s for 4 agents, and 84 s for 5 agents to reach the destination. These values clearly demonstrate the trend of the minimization of the total average travel time in FLDQN and the dependency of the overall system on the number of agents.

In Figure 7, the results for DQN and FLDQN over the number of agents illustrate the independence of DQN and the dependency in FLDQN. FLDQN shows a trend of gradually reducing the total average travel time as the number of agents increases, demonstrating that increasing the number of agents improves the likelihood of minimizing the travel time. The improvement in FLDQN is attributed to inter-agent weight sharing, where all agents benefit from a single network learned during training. Agents leverage each other’s experiences through our parameter-sharing scheme, which allows simultaneous training with unique observations for each agent. This shared learning process involves agents with diverse local experiences, such as different traffic patterns or environmental conditions across runs. These differences in local experiences contribute to the higher standard deviation in FLDQN compared to DQN, as the variability between agents’ environments leads to greater variation in performance outcomes. However, the higher standard deviation also highlights the potential for discovering better policies for more effective travel time minimization, as agents can draw on a broader pool of experiences. As a result, FLDQN demonstrates the potential for enhanced travel time minimization as the number of agents increases, underscoring the effectiveness of inter-agent cooperation and shared learning.

To access whether parameter sharing in FLDQN contributes to a reduction in running time, defined as the time until the agents finish the predefined episodes, we evaluate the overall running time for both DQN and FLDQN.

**Figure 8:**
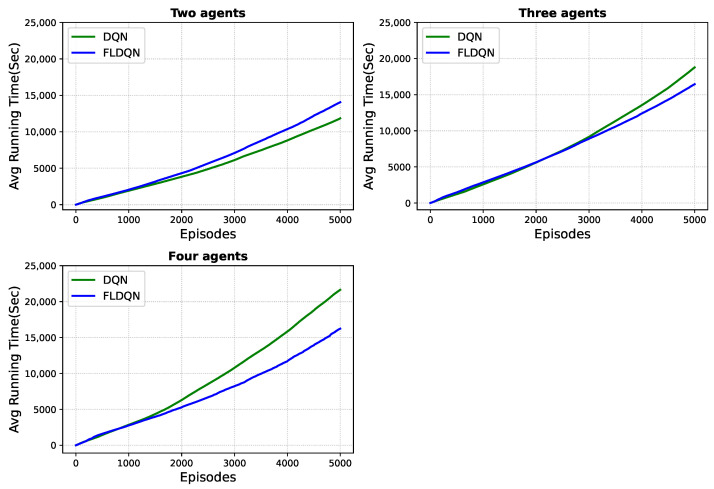
A comparative analysis of average running time between DQN and FLDQN. The results underscore the efficiency gains achieved by FLDQN through parameter sharing, leading to reduced computation costs and accelerated training.

As depicted in Figure 8, FLDQN outperforms DQN in terms of total running time. In a two-agent setup, DQN requires 11,800 s to complete 5000 episodes, whereas FLDQN takes 14,000 s. When the number of agents is increased to three, DQN consumes 18,800 s, while FLDQN takes 16,400 s for 5000 episodes. With four agents, the running time of DQN is 21,600 s, whereas FLDQN takes only 16,200 s for 5000 episodes. This trend highlights that as the number of agents increases, FLDQN exhibits a decreasing average running time compared to DQN, where each agent runs independently. The efficiency gains can be attributed to the weight-sharing mechanism employed in FLDQN, where all agents utilize the weights of a single network learned during the training process. This approach reduces the learning time by minimizing the number of parameters that need to be learned as discussed previously. Our proposed approach assumes a common set of actions among agents while providing each agent with unique observations and hidden states. Furthermore, agents can be trained concurrently, thanks to the parameter-sharing scheme, which takes individual observations into account while leveraging the collective experiences of all agents. This feature clearly positions our suggested approach to lower computation costs and accelerate the training process.

In conclusion, DQN operates independently, with each agent working in isolation without the ability to influence or share information with others. This inherent lack of inter-agent communication and parameter sharing in DQN can result in inefficiencies during the learning process. As a consequence, each agent may experience varying convergence times, potentially leading to instances where some agents take more or less time than others to complete their learning objectives. Unlike cooperative approaches, such as FLDQN, where agents can share experiences and insights through parameter sharing, the independent nature of DQN may limit its ability to benefit from collaborative learning dynamics, impacting its overall efficiency and adaptability in multi-agent scenarios. To conclude, Figure 7 and Figure 8 suggest that increasing the number of agents in FLDQN further enhances the overall system performance.

### 5.3. Travel Time Comparison After Learning

RL algorithms are typically assessed using two key metrics: “evaluation after learning” which evaluates a fixed policy after training, and “evaluation during training” calculated throughout the training process [49]. The evaluation of RL agents is crucial, especially when implemented in real-world scenarios, where challenges may arise. In this section, we focus on “evaluation after learning”, as it aligns with our goal of identifying reward faults, ensuring the models learn the intended behavior. Testing aims to evaluate agent correctness, addressing reward faults (not reaching intended rewards) and functional faults (taking actions leading to unsafe states). Based on the special reward mechanism, our scenario prioritizes reward faults, evaluating model learning against expectations. To assess agent performance, we save the model weights for both DQN and FLDQN during training. After learning, we load these weights to evaluate their learning outcomes. Testing involved 1000 episodes in the SUMO simulation environment for comprehensive evaluation.

**Figure 9:**
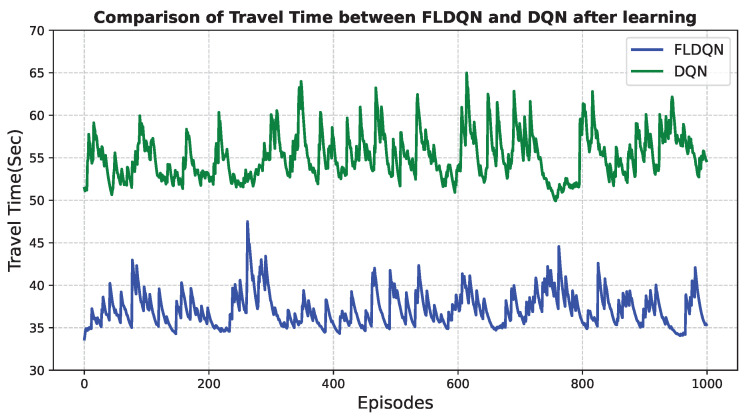
Comparison of travel time after learning in DQN and FLDQN: From this plot, it is clear that FLDQN outperforms DQN, demonstrating more efficient learning of policies.

Results depicted in Figure 9 demonstrate that FLDQN outperforms DQN, with FLDQN achieving an average travel time of 33.6 s, compared to 51.4 s for DQN. This equates to a 34.6% reduction in travel time, calculated as in Equation (9):

(9) $$\mathrm{Percentage}\;\mathrm{Reduction}=\frac{\mathrm{DQN}\;\mathrm{time}-\mathrm{FLDQN}\;\mathrm{time}}{\mathrm{DQN}\;\mathrm{time}}\times 100$$

$$=\frac{51.4-33.6}{51.4}\times 100=34.6\%$$

This significant difference highlights the superior learning capabilities of the proposed FLDQN model. The substantial improvement in travel time suggests its potential for real-world applications where efficient traffic management is crucial. In conclusion, the superior performance of FLDQN can be attributed to its unique strengths compared to DQN. The weight sharing mechanism and collaborative learning approach enable agents to leverage collective knowledge and improve individual decision-making, resulting in significantly faster travel times and demonstrating the potential of FLDQN for effective traffic management applications.

### 5.4. Discussion and Implications

The results presented in this study demonstrate the effectiveness and robustness of the proposed FLDQN algorithm in solving travel time minimization problems within MARL scenarios. This work addresses the limitations of traditional single-agent RL by introducing a cooperative, FL-based multi-agent approach. In FLDQN, agents are able to share their experiences through a federated server in an efficient manner. This allows agents to influence each other’s policies and make optimal decisions while maintaining data privacy, as only model weights are exchanged rather than raw data. This characteristic makes FLDQN particularly suitable for scenarios requiring collaboration and knowledge sharing, especially where data privacy is crucial. The obtained results indicate that with more agents involved, the better and more robust the overall system becomes. This is because agents can collectively explore the environment more effectively, accelerating the exploration process for other agents. Such cooperation fosters a faster convergence towards optimal decision-making and enhances the system’s ability to adapt to dynamic conditions.

To validate the proposed algorithm, we employ the SUMO simulation environment, which has been widely validated in the literature [22,37,40,41] for its ability to simulate scenarios closely resembling real-world traffic conditions. Based on these studies, SUMO is selected as an appropriate tool to demonstrate the feasibility and effectiveness of our methodology. It is worth noting that FLDQN has the potential to be generalized and applied to other real-world scenarios beyond traffic congestion. Any setting where multiple agents require cooperative decision-making, secure data handling, and shared learning could benefit from this approach. Furthermore, this study paves the way for future research to explore the applicability of FLDQN in domains such as smart fleet management, supply chain management, and collaborative robotics, where the principles of FL can be extended.

## 6. Conclusions and Future Scope

This paper introduced FLDQN, an innovative Federated Learning-based Deep Q-Learning Network designed to tackle the challenge of agent cooperation in MARL by minimizing travel time in dynamic traffic environments. Motivated by the absence of efficient and privacy-preserving solutions for agent cooperation, FLDQN utilizes FL to foster collaboration through privacy-preserving weight sharing. The proposed methodology seamlessly integrates FL, DRL, and the SUMO simulator into a cutting-edge framework that promotes effective agent cooperation. Multiple intelligent agents equipped with individual DQN models navigate the traffic networks generated by SUMO. Through cyclic weight exchanges coordinated by a central server, agents benefit from shared experiences and collective knowledge. Extensive experiments showcase the prowess of FLDQN in reducing both travel time and running time during training, surpassing the performance of the non-cooperative approach. The results demonstrate an impressive 34.6% average improvement in minimizing total travel time. Furthermore, the findings reveal a positive correlation between increased agent counts and enhanced system performance, underscoring the critical impact of inter-agent cooperation. In summary, the results demonstrated in this work provide a new perspective on the importance of efficient agent cooperation in MARL scenarios. FLDQN provides groundbreaking insights into achieving effective multi-agent cooperation, highlighting knowledge sharing as the catalyst for improved decision-making. The proposed solution offers adaptable traffic optimization abilities, showcasing promising real-world applications. Looking ahead, our future work aims to enhance FLDQN by minimizing communication costs and considering other factors such as tunnels, bridges, and multi-level junctions to further optimize the efficiency of the cooperative multi-agent system.

## Figures and Tables

**Table 1:** Table displaying the comprehensive set of hyperparameters utilized in our proposed FLDQN experiments.

Hyperparameters	Values
Fraction-fit	1.0
Min-fit-clients	5
Min-available-clients	5
Episodes	100
Rounds	50
Discount-factor	0.99
Learning-rate	0.001
Epsilon	1.0
Epsilon-Decay	0.95
Epsilon-min	0.05
Batch-size	32
Train-starter	200
Reply-Memory	100,000

## Data Availability

The data supporting the reported results can be generated by running the experiments. The source code can be accessed at https://github.com/nclabteam/FLDQN (accessed on 23 January 2025).
